# Molecular Characterization and Moxifloxacin Susceptibility of *Clostridium difficile*

**DOI:** 10.3390/antibiotics8030118

**Published:** 2019-08-12

**Authors:** Sarah Mizrahi, Zohar Hamo, Maya Azrad, Avi Peretz

**Affiliations:** 1 The Azrieli Faculty of Medicine, Bar-Ilan University, Safed 1311502, Israel; 2 Clinical Microbiology Laboratory, Baruch Padeh Medical Center, Poriya, affiliated with the Faculty of Medicine, Bar Ilan University, Safed 1311502, Israel

**Keywords:** *Clostridium difficile*, GenoType CDiff kit, moxifloxacin, ribotype027, ribotype078, *gyrA*

## Abstract

In recent years, the incidence and severity of *Clostridium difficile* infections has increased. Additionally, resistance of *C. difficile* to frequently used antibiotics is rising. To improve our understanding of *C. difficile*, there is a need for molecular characterization of different strains and antibiotic resistance testing. We investigated the efficacy of GenoType CDiff kit (Hain Lifesciences) in identification of *C. difficile* and its various strains in northern Israel. The kit involves a molecular assay that detects *C. difficile* from stool samples or colonies and identifies the different strains and mutations in the *gyrA* gene that cause moxifloxacin resistance. Forty-nine *C. difficile* positive samples were examined by the kit following DNA extraction from both colonies and stool. The identification rate (95.9%) of *C. difficile* was much higher when DNA was extracted from colonies, compared to extraction from stool (46.9%). Low frequencies of ribotype027 strain (2%) and of ribotype078 strain (4%) were found. There was a high concordance between genotype (mutation in *gyrA*) and phenotype (Etest) for moxifloxacin resistance (Kappa = 0.72). A high percentage of moxifloxacin-resistant strains was found. Our findings indicate that the GenoType CDiff kit is very effective in characterization of *C.* *difficile* strains and less effective for identification of *C. difficile* directly from stool samples.

## 1. Introduction

*Clostridium difficile* is a gram-positive, obligate anaerobic bacillus that is the predominant agent of antibiotic-associated diarrhea, and is associated with serious morbidity and potential mortality [1,2,3]. A recent review that evaluated the global burden of *Clostridium difficile* infection (CDI) has found that the overall rate of healthcare- associated CDI was 2.24 per 1000 admissions/year [4].

CDI is a toxin-mediated disease, mainly caused by the production of two major enterotoxins, toxin A and B [5]. Following their release, the toxins catalyze the inactivation of small GTPases (such as Rho, Rac, and Ras), which results in cytoskeletal disorganization, hemorrhage, and the release of fluid into the intestinal tract, causing the watery diarrhea that is characteristic of CDI [6]. 

The toxin genes, *tcdA* (toxin A) and *tcdB* (toxin B), are located within a pathogenicity locus (PaLoc), alongside genes encoding for negative (*tcdC*) and positive (*tcdD*) regulators of toxin transcription. One of the most virulent strains, ribotype 027/NAP1 *C. difficile*, has a deletion in the *tcdC* locus, which results in increased production of toxins A and B and in increased disease severity [7]. Some *C. difficile* strains produce a third toxin, the binary toxin, which is found in some epidemic strains such as ribotype 027 and 078 and enhances the severity of CDI disease [8].

In past years, there was an increase in CDI incidence and severity in both hospital and community settings. This increase was correlated with the emergence of hypervirulent *C. difficile* strains ribotype027 and ribotype078 [9,10,11]. Routine diagnosis of *C. difficile* usually does not include characterization of the specific strain. Also, the distribution of different *C. difficile* strains in Israel is not well known [12].

The clinical expression associated with CDI is mainly attributable to the epithelial cell damage and inflammation caused by *C. difficile* toxins. Disease symptoms include mainly diarrhea, abdominal pain, and fever. During severe infections, pseudomembranous colitis, toxic megacolon, or colonic perforation can occur, sometimes leading to death [13]. 

Risk factors for CDI include age above 65, hospitalization in the intensive care unit (ICU), inflammatory bowel diseases, chemotherapy, and previous antibiotic therapy, which is the major risk factor [14,15,16,17]. Antibiotic administration disrupts and changes the normal intestinal microbiota, resulting in germination and proliferation of *C. difficile* spores [17]. In fact, 1–3% of hospitalized patients receiving antibiotics become infected with *C. difficile* and about 25% of them will experience recurrent infections [18]. 

CDI therapy includes cessation of prior antibiotic therapy and treatment with vancomycin in most cases [19]. However, the use of vancomycin may lead to development of resistance in other bacteria, such as vancomycin-resistant *Enterococcus* (VRE) [20]. In severe and recurrent cases, fecal microbiota transplantation (FMT) that restores the normal fecal microbiota is efficient [21]. 

Similar to other bacteria, *C. difficile* has developed antibiotic resistance. Several strains are resistant to a variety of antibiotics such as aminoglycosides, lincosamides, macrolides, penicillins, cephalosporins, and fluoroquinolones, that are commonly used in clinical treatment [22]. In fact, this resistance increases the risk of developing CDI and can lead to treatment failure and recurrence. 

*C. difficile* has also developed resistance to specific antibiotics that are used to treat CDI. For example, a study carried out in the United States that examined 925 isolates found that resistance rates were 3.6%, 17.9%, and 33.5% for metronidazole, vancomycin, and moxifloxacin, respectively [23]. A former study conducted by our group among 81 *C. difficile* isolates from northern Israel has found that 7.4% of the isolates were resistant to vancomycin, 4.9% to metronidazole, 21% to moxifloxacin, and 1.23% to tigecycline [24]. The antibiotic resistance is attributed to multiple mechanisms, including transposons (mobile genetic elements) and various genetic mutations. For example, resistance to fluoroquinolones is usually caused by a mutation in the *gyrA* gene that encodes for DNA gyrase, an enzyme that is involved in bacterial replication [13]. The fluoroquinolone family inhibits this enzyme and therefore mutations in *gyrA* subunits (*gyrA* MUT1A, *gyrA* MUT1B) confer resistance to moxifloxacin.

Moxifloxacin belongs to the quinolone family that is not used for the treatment of *C. difficile*. However, a high rate of resistance to this antibiotic could predict an infection with a virulent strain or therapeutic failure [25].

Unlike other bacterial infections that are diagnosed by culture, the detection of CDI is based mainly on indirect methods. The main reason for this is the difficulties in cultivating the bacterium due to special incubation conditions and the need for selective growth substrates [26]. CDI diagnosis is usually carried out using enzyme-linked immunosorbent assay (ELISA) or molecular biology for detection of toxin presence or bacterial DNA from stool [27,28]. Detection of *C. difficile* antigen, glutamate dehydrogenase (GDH), via latex agglutination in stool is used; however, this tool does not distinguish between nontoxigenic and toxigenic strains [29]. The cell culture cytotoxicity assay (CCCA) has a high specificity (99%), but is very expensive and time-consuming (48–72 h). Using this technique, a liquid phase containing the toxins is separated from the stool sample, and incubated with a cell culture. The toxins, once present, cause morphological changes in the cell [30]. The ELISA for toxin A or toxin B is usually used despite the test’s lower sensitivity (70–90%), given the fact that is cheaper and less technically demanding [31]. The test detects the presence of toxins by monoclonal or polyclonal antibody binding. 

*C. difficile* identification can be performed by PCR-based assays that detect the presence of *C. difficile* genes. One such commercial kit is the GeneXpert *C. difficile* assay (Cepheid, Sunnyvale, CA, USA) that detects three gene targets: binary toxin, toxin B, and *tcdC* deletion [28]. 

In this study, we used GenoType CDiff kit (Hain Lifesciences)—a molecular assay that identifies *C. difficile* from stool or colonies and can differentiate nonpathogenic, pathogenic, and hypervirulent strains (such as ribotype027). The kit also identifies the two most common mutations that lead to moxifloxacin resistance. Most molecular assays that identify different strains are laborious and time-consuming. Some of these assays involve sequencing, which is still costly. The GenoType CDiff kit offers a quite simple protocol, which enables strain identification and antibiotic resistance detection within 6 h. We aimed to compare the kit efficiency for identification of *C. difficile* from bacterial colonies vs. directly from stool samples, to characterize the different strains, and to compare two susceptibility determination methods for moxifloxacin (GenoType CDiff kit and Etest).

## 2. Results

### 2.1. A Comparison Between the Two Methods for DNA Extraction

A total of 49 samples were examined for *C. difficile* presence by two methods. These samples were collected from patients hospitalized at Padeh Poria medical center. A total of 47 (95.92%) samples were positive for *C. difficile* by GenoType CDiff when the DNA was extracted from colonies, compared to 23 (46.94%) positive samples when the DNA was extracted was from stool (Table 1). 

**Figure 1:**
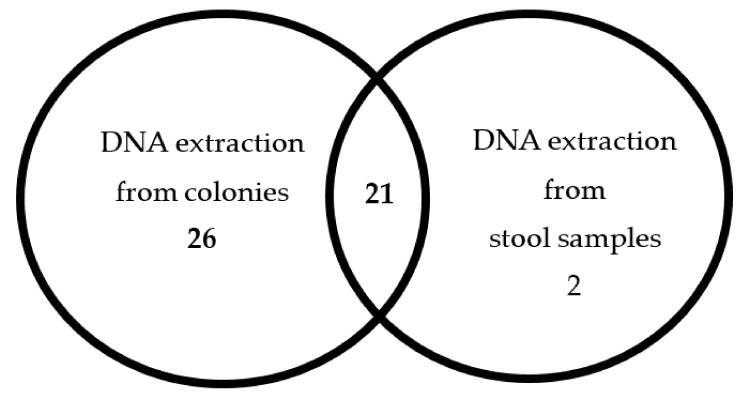
Number of positive samples for *C. difficile* detected by each method for DNA extraction.

Twenty-one (42.8%) samples were found positive by the kit when extracted either from stool or colonies (Figure 1); 26 (53.1%) samples were detected positive only when DNA was extracted from colonies; 2 (4.1%) samples were detected positive only when DNA extraction was from stool samples.

### 2.2. Genotypes Detected by Genotype CDiff

**Figure 2:**
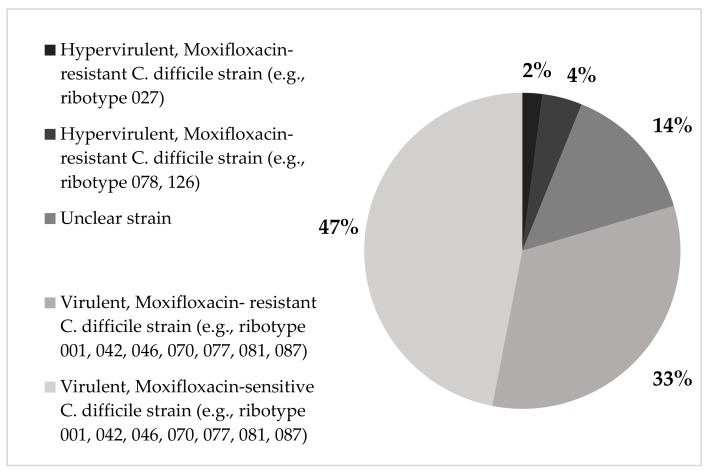
Distribution of different *C. difficile* strains.

Twenty-three samples (47%) were characterized by the GenoType CDiff as virulent moxifloxacin-sensitive *C. difficile* strains (i.e., ribotypes 001, 042, 046, 070, 077, 081, 087). Sixteen samples (33%) were identified as virulent, moxifloxacin-resistant *C. difficile* strains (i.e., ribotypes 001, 042, 046, 070, 077, 081, 087); two samples (4%) as hypervirulent moxifloxacin-resistant *C. difficile* strains (ribotypes 078, 126). Only one sample (2%) was characterized as a hypervirulent *C. difficile* ribotype 027. For 7 samples (14%) the *C. difficile* strain was unclear (Figure 2).

### 2.3. The Correlation of C. difficile Strains with Infection Acquisition and Disease Severity

Out of 28 hospital-acquired *C. difficile* isolates (HA-CD), 12 (42.8%) were virulent and moxifloxacin-sensitive, and 11 (39.3%) isolates were moxifloxacin-resistant. Among the 21 community-acquired *C. difficile* isolates (CA-CD), the most frequent strains were virulent moxifloxacin-sensitive *C. difficile* (11 isolates, 52.4%). One isolate (3.6%) of the HA-CD group was hypervirulent ribotype027 *C. difficile* strain, compared to none of CA-CD (Table 2). However, no statistically significant correlation was found between infection acquisition and *C. difficile* ribotypes (*p* = 0.65).

Among the 35 patients with mild disease, the most frequent isolated strains were virulent, moxifloxacin-sensitive and moxifloxacin-resistant strains (17/35 and 11/35 isolates, respectively). In addition, the only isolate that was characterized as hypervirulent *C. difficile* ribotype027 strain in this study was isolated from a patient with a mild disease. In the moderate disease group (N = 11), the most frequent strains were virulent, moxifloxacin-sensitive (6/11 samples). Out of three patients with severe disease, all isolates were virulent and moxifloxacin-resistant (Table 2). However, no statistical significance was found for the correlation between disease severity and *C. difficile* ribotypes (*p* = 0.3).

### 2.4. Comparison of Antibiotic Susceptibility to Moxifloxacin by MIC Breakpoints (Phenotype) and Gene Expression (Genotype)

Forty-nine samples were defined as positive for *C. difficile* by at least one of the two DNA extraction methods. These samples were also tested by the kit for distribution of wild type and most common mutant alleles of the *gyrA* gene. The *gyrA* gene encodes for DNA gyrase, an enzyme that is involved in bacterial replication. Antibiotics belonging to the Fluoroquinolone family inhibit this enzyme. Mutations in *gyrA* subunits (*gyrA* MUT1A, *gyrA* MUT1B) confer resistance to moxifloxacin. 

**Figure 3:**
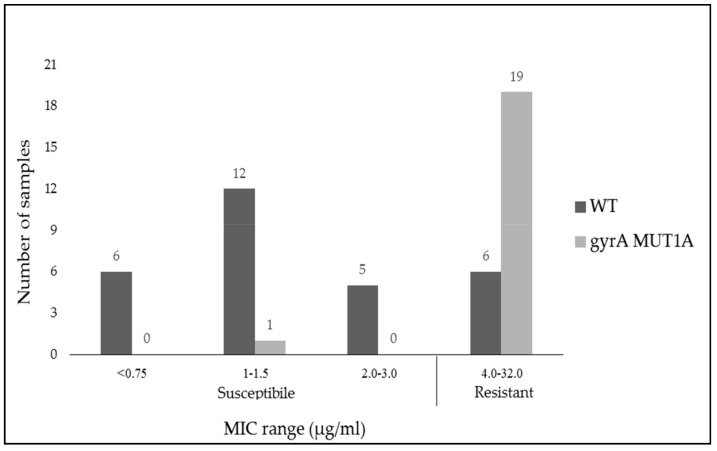
Sample distribution by MIC ranges and allele appearance for moxifloxacin antibiotic. * WT = Wilt type; MIC = Minimum inhibitory concentration.

Figure 3 presents the Minimum inhibitory concentration (MIC) values of the different isolates to moxifloxacin. 

The *gyrA* Wilt-Type (*gyrA* WT) gene was identified in 29 samples; 6 of them had an MIC above 4 µg/mL, which points to moxifloxacin resistance (Table 3). Mutations in *gyrA* were found in 20 samples (all of them *gyrA* MUT1A). One mutant strain was susceptible to moxifloxacin according to its MIC result.

Twenty-five (51%) samples were found to be resistant by Etest, 6 of them were with *gyrA* WT allele; 24 (49%) samples were found to be sensitive by Etest, one of them was with *gyrA* MUT1A. The *gyrA* MUT1B mutation was not found in this study. A comparison of GenoType CDiff (KIT) vs. Etest MIC (reference) revealed a sensitivity of 76% [95% CI 56.6–88.5] and specificity of 95.8% [95% CI 79.8–99.3]. Agreement level was 85.7%, with kappa coefficient of 0.72. 

## 3. Discussion

In this study, we examined the efficacy of the GenoType CDiff kit for identification of *C. difficile* from stool and colonies and for characterization of different strains in north Israel.

DNA extraction from colonies resulted in a high correlation (96%) with the reference method (GeneXpert). A similar diagnostic sensitivity (86%) was found in another study [32]. In contrast, DNA extraction directly from stool resulted in a much lower correlation (9.5%). A possible explanation for this can be the low quantity/quality of DNA that did not allow efficient amplification. 

Two samples that were identified as negative when DNA was extracted from colonies were identified as positive when DNA was extracted from stool. These results are surprising, since we would have expected that samples taken directly from a colony would contain higher amounts of *C. difficile* DNA. A possible explanation is that the DNA was extracted from a colony that was not *C. difficile*, although the medium was selective and the sampled colonies were similar in morphology to *C. difficile*. Hence, it is important to perform gram staining and further identification of the colony with more advanced tools, for example using the MALDI-TOF spectrometry system.

We found that all the samples contained both toxin genes. In a study published in 2017, in which 411 positive samples for *C. difficile* were examined, 68.6% of the samples contained both genes for toxins, 23% contained the toxin B gene, and 8.5% did not contain the toxin genes at all [33].

In the current study, 6% of the strains contained *cdtA* and *cdtB* genes that encode the binary toxin and deletion in toxin regulator gene *tcdC* that encodes the anti-sigma factor that is involved in downregulation of toxin A and toxin B [34]. These strains are characterized as hypervirulent. In this category we can find the ribotype027 strain that has a high resistance rate to moxifloxacin [35,36] and ribotype078 [11]. Two percent of the hypervirulent strains in this study were ribotype027. This is a low rate compared with other reports in the world and in Israel in particular [12,36]. Perhaps ribotype027 strain is uncommon in north Israel. Four percent of the hypervirulent strains were ribotype078 strain, consistent with other regions that reported an increase in this strain [36]. However, this percentage is not consistent with a study conducted in Israel, where the prevalence was 1.9% [12]. Again, it is possible that different geographic areas are characterized with a different prevalence of strains.

We found that 57.2% of CDI cases were hospital acquired (HA) and 42.8% were community acquired (CA). This result is consistent with studies showing that prevalence of CA-CDI is increasing [37]. In the current study, ribotype027was present only in HA-CDI (3.6% of HA-CDI). A higher percentage of this ribotype was found in HA-CDI compared with CA-CDI in 2016 [38]. However, a study conducted in Canada found that the ribotype027 was more prevalent in CA-CDI than in HA-CDI [35].

There was a high frequency of moxifloxacin-resistant *C. difficile* strains in HA-CDI (39.3%). Also, we found a significant percentage of moxifloxacin-resistant *C. difficile* strains in CA-CDI (23.8%). The hypervirulent ribotype078 was found in a higher percentage in CA-CDI (4.8%) compared to HA-CDI (3.6%). In contrast, in another study conducted in Israel ribotype078 strain was more common among HA-CDI [38]. Other studies showed a high frequency of ribotype078 in CA-CDI [11,39]. However, there was no statistically significant association between infection source and different strains.

The study also examined the association between disease severity and different strains of *C. difficile*, but there was no statistically significant association. Moreover, the infections caused by ribotype027 and hypervirulent moxifloxacin-resistant strains (078, 126) were classified as mild. In addition, all severe CDI cases were caused by *C. difficile* strains that had a genetic moxifloxacin resistance. Several studies have found no association between disease severity and ribotype 027 [40,41,42].

Regarding the correlation between genotype and phenotype of moxifloxacin resistance, we found a high concordance between the genotype and phenotype (Kappa = 0.72). One isolate that carried the mutation showed moxifloxacin susceptibility phenotypically. It is possible that the bacterium does not necessarily express the mutated allele. Six isolates that showed phenotypical resistance did not carry any mutation. A possible explanation is that there are other mutations in *gyrA* or *gyrB* genes that are not identified by the kit [43]. 

The percentage of samples found to be moxifloxacin resistant according to phenotype and genotype was 51% and 40.8%, respectively. This rate is higher than the resistance rates found in other studies; for example a European study from 2005 found that 37.5% of tested strains were moxifloxacin resistant (by Etest) [44]. In a study from Shanghai, 46.4% of the isolates were moxifloxacin resistant [45]. The high resistance rate to moxifloxacin is possibly related to the increased use of fluoroquinolones in recent years [46]. In a study published in 2003, a link was found between previous intake of fluoroquinolones and the development of moxifloxacin-resistant *C. difficile* strains. However, most patients in this study took non respiratory fluoroquinolones and none of them took moxifloxacin [47]. In another study, it was found that exposure to levofloxacin and moxifloxacin in vitro often results in selection for *gyrA* and *gyrB* mutant strains in previously susceptible strains [48]. Due to the high prevalence of moxifloxacin resistance, which is a predictor of hypervirulent bacteria, there may be a need to consider better antibiotic stewardship of fluoroquinolones.

## 4. Materials and Methods 

### 4.1. Study Population

Forty-nine stool samples were collected from CDI patients at the Poriya Baruch Padeh Medical Center, from November 2015 to May 2017. Specimens were analyzed by the Xpert *C. difficile* Assay (Cepheid, Sunnyvale, CA, USA), for rapid identification of Toxin B, Binary Toxin, and *tcdC* deletion presence.

An institutional review board, POR 0003-15, approved the study and informed consent was obtained from participants.

### 4.2. Disease Severity Score and Epidemiologic and Clinical Data Collection

Disease severity was determined according to Severity Score Index [49], as previously described [50]. The following data was retrospectively collected from medical records: age, gender, community versus hospital acquired CDI (diarrhea started after 48 hours from hospitalization), and death during hospitalization.

### 4.3. C. difficile Susceptibility to Moxifloxacin

Stool samples were cultured on chromID^TM^ *C. difficile* (bioMérieux, Durham, NC, United States) growth medium and then incubated at 37 °C in anaerobic conditions for 48 h. *C. difficile* colonies appear asymmetric and black colored [50]. Isolated colonies were suspended for generation of 1 McFarland turbidity. The inoculum was sub cultured on Brucella Blood Agar (Becton Dickinson, Heidelberg, Germany), supplemented with hemin and vitamin K, and an Etest strip for moxifloxacin (bioMérieux, Durham, NC, United States) was added. The agar plates were incubated in anaerobic conditions at 37 °C for 24 h. Minimum inhibitory concentration (MIC) determination was performed in accordance with the European Committee on Antimicrobial Susceptibility Testing (EUCAST) guidelines; *C. difficile* isolates were considered resistant when MIC to moxifloxacin was above 4 µg/mL [12].

### 4.4. DNA Extraction

DNA was extracted both from isolated colonies and directly from stool samples. Five isolated colonies were suspended in 30 µL DNA-free water and incubated at 100 °C for 10 min. Following centrifugation (12,000 rpm, 1 min), the pellet was discarded and supernatant was used for PCR amplification. DNA was also extracted from 200 mg stool using the QIAamp Fast DNA Stool Mini Kit (QIAGEN, Hilden, Germany) according to the manufacturer’s instructions.

### 4.5. Molecular Identification Using GenoType CDiff kit

We used the GenoType CDiff kit (Hain Lifesciences, Nehren, Germany) according to the manufacturer’s recommendations. Briefly, following DNA amplification, hybridization protocol was performed at 45 °C. The membrane strips are coated with specific probes complementary to the following genes: toxin genes (*tcdA*, *tcdB*), binary toxin genes (*cdtA*, *cdtB*), deletion on regulator gene *tcdC* (18 bp deletion, 39 bp deletion, and single base deletion in position 117), and *gyrA* mutation (*gyrA* MUT1A, *gyrA* MUT1B). The hybridization bands were detected as visible lines on the membrane strips. 

### 4.6. Statistical Analysis

The agreement between each method of extraction and the Xpert *C. difficile* Assay was calculated as the percentage of isolates that had the same result by the two methods.

A Chi-square test was applied for analyzing the correlation between different ribotypes and infection acquisition as well as the correlation of different ribotypes and disease severity.

For analysis of sensitivity and specificity of GenoType CDiff kit for moxifloxacin resistance (by genotype), we used the Etest as the reference method for antibiotic susceptibility (by phenotype). Specimens that were found resistant or sensitive by the Etest were defined as “True Resistant” or “True Sensitive”, respectively. Agreement level was analyzed by calculating Kappa coefficients, with the acceptable following range: r < 0 poor agreement; 0 ≤ r ≤ 0.2 slight agreement; 0.21 ≤ r ≤ 0.4 fair agreement; 0.41 ≤ r≤ 0.6 moderate agreement; 0.61 ≤ r ≤ 0.8 substantial agreement; and 0.81 ≤ r ≤ 1 almost perfect agreement.

## 5. Conclusions

We found the GenoType CDiff (Hain Lifesciences) kit is very effective in the characterization of different *C. difficile* strains when DNA is extracted from culture. However, the kit is less effective for identification of *C. difficile* directly from stool samples. Furthermore, among the *C. difficile* we isolated in northern Israel, we found high levels of moxifloxacin resistance. Further larger scale studies need to be conducted to examine the distribution of the various *C. difficile* strains and their antibiotic resistance in Israel. Additionally, future studies may include strain ribotyping in order to study strain distribution in Israel and to reveal whether there is correlation between GenoType CDiff and ribotyping.

## Figures and Tables

**Table 1:** Comparison between identification of *C. difficile* by GenoType CDiff from stool samples vs. culture samples.

Result	GenoType CDiff (*n* = 49)	
	Culture *n* (%)	Stool *n* (%)
Positive	47 (96)	23 (47)
Negative Percent of Agreement (compared with GeneXpert Results)	2 (4)	26 (53)
	95.9 [95% CI *: 86.0, 99.5]	46.9 [95% CI: 32.5, 61.7]

* CI = confidence Interval.

**Table 2:** Distribution of different *C. difficile* strains in correlation with Infection Acquisition and Disease Severity.

*C. difficile* Strain	Infection Acquisition		Disease Severity				
	Community (Total N = 21) N (%)	Hospital (Total N =28) N (%)	*p*-Value	Mild (Total N= 35) N (%)	Moderate (Total N = 11) N (%)	Severe (Total N = 3) N (%)	*p*-Value
Virulent, Moxifloxacin-sensitive	11 (52.4)	12 (42.8)	0.65	17 (48.6)	6 (54.5)	0 (0)	0.3
Virulent, Moxifloxacin-resistant	5 (23.8)	11 (39.3)		11 (31.4)	2 (18.2)	3 (100)	
Hypervirulent, Moxifloxacin-resistant (078, 126)	1 (4.8)	1 (3.6)		2 (5.7)	0 (0)	0 (0)	
Hypervirulent ribotype027	0 (0)	1 (3.6)		1 (2.9)	0 (0)	0 (0)	
Unclear strain	4 (19)	3 (10.7)		4 (11.4)	3 (27.3)	0 (0)	

**Table 3:** Correlation of moxifloxacin resistance in accordance with GenoType CDiff kit and Etest.

		Etest Result	
		Sensitive	Resistant
GenoType CDiff	**Resistant** (*gyrA* MUT1A)	1 (2.1%)	19 (38.7%)
	**Sensitive** (*gyrA* WT)	23 (47%)	6 (12.2%)
